# Low free triiodothyronineis predicts worsen neurological outcome of patients with acute ischemic stroke: a retrospective study with bioinformatics analysis

**DOI:** 10.1186/s12883-019-1509-x

**Published:** 2019-11-05

**Authors:** Shanchao Zhang, Xia Zhao, Shan Xu, Jing Yuan, Zhihua Si, Yang Yang, Shan Qiao, Xuxu Xu, Aihua Wang

**Affiliations:** Department of Neurology, Shandong Provincial Qianfoshan Hospital, the First Hospital Affiliated with Shandong First Medical University, NO.16766 JingShi Road, Jinan, 250014 Shandong China

**Keywords:** Stroke, Triiodothyronineis, Prognosis, Bioinformatics analysis

## Abstract

**Backgroud:**

Patients with acute ischemic stroke (AIS) often experience low serum free triiodothyronine (FT3), but the association of low FT3 with stroke severity, subtype and prognosis has not yet been thoroughly studied, and the molecular events underlying these clinical observation were also unclear.

**Methods:**

We retrospectively collected 221 cases of AIS and 182 non-AIS cases with detailed clinical data from our department. FT3 concentrations were measured on admission to predict functional outcome within 3 months using multivariable models adjusted for other risk factors. Receiver operating characteristic (ROC) curves were calculated to define the best cutoff value of FT3 of stroke severity, subtypes and neurological outcome. Gene set enrichment, pathway mapping and network analyses of deferentially expressed genes (DEGs) were performed.

**Results:**

FT3 was significantly decreased in AIS patients with National Institutes of Health Stroke Scale (NIHSS) > 3 and 3-months modified Rankin Scale (mRS) > 2. The cut-off value of FT3 for NIHSS on admission was 4.30 pmol/L. Also, FT3 level was significantly lower in large artery atherosclerosis (LAA) group and cardioembolism (CE) group than that in small vessel occlusion (SVO). FT3 value served as an independent predictor for neurological outcomes for which the cut-off value of FT3 was 4.38 pmol/l. Gene ontology (GO) analysis showed that the biological function of DEGs was mainly enriched in multicellur organism, neuron differentiation and cellular response to hypoxia. The cellular components were involved in extracelluar region, exosome and matrix, and the molecular functions were transcriptional activator activity, DNA binding and nuclear hormone receptor binding. Signal pathways analysis was indicative of neuroactive ligand-receptor interaction, thyroid hormone signaling pathway, and protein digestion and absorption these DEGs were involved in. Six related gene were identified as hubs from the protein-protein interaction (PPI) networks. Three modules were selected from PPI, of which MMP4, ADRA2C and EIF3E were recognized as the seed genes.

**Conclusions:**

Low FT3 value on admission was associated with stroke severity, subtype and prognosis. In addition, DEGs identified from bioinformatics analysis are likely to be candidates for elucidating clinical outcomes with low FT3, and provide us with therapeutic targets for improving stroke prognosis.

## Introduction

Neuroendocrine system could be significantly altered in acute stroke status. Most of the clinical studies related to neuroendocrine findings in AIS focus on the hypothalamic-pituitary-adrenocortical axis and related stress mechanism [1, 2]. It also has reported that during diverse cardiovascular critical statuses, thyroid gland metabolism also changed [3]. This problem was defined as non-thyroidal illnesssyndrome (NTIS), also called low T3 syndrome, in which abnormalities of thyroid hormones occur due to non-thyroidal system disorder [4]. A growing number of studies indicate that the low T3 syndrome is a common complication in the patients with AIS that correlated with greater neurological impairment upon presentation, and greater disability and mortality among stroke patients [5–7]. Moreover, the role of low T3 in the prognosis of stroke patients is independent from well-established clinical prognostic indicators, suggesting that the low T3 syndrome can exert prognostic value above and beyond currently used stroke prediction models [8]. In contrast, some studies have represented conflicting results that displayed a protective association between subclinical hypothyroidism and better outcomes following stroke [9, 10]. In addition to the inconsistent conclusion, It is not well recognized whether there is any association between FT3, the bioactive form of T3, and subtypes of stroke on admission. Thus, one of our study goals is to analyze the role of low FT3 on stroke prognosis and the association between low FT3 and clinical types of AIS.

Thyroid hormones (THs), including triiodothyronine (T3) and thyroxine (T4), are essential for brain development, morphogenesis and maturation processes [11]. Studies in animal stroke model have shown that T3 has therapeutic effects on cerebral ischemic stroke by increasing the neurotrophic factors and in vitro reduction of inflammation and restoration of neurotrophin expression, which lead to better neurological function [12, 13]. Despite the mechanisms depicted by the above studies, the detailed signaling pathways by which T3 affects functional outcome of stroke patients remain to be elucidated. Therefore, it is crucial to uncover these signaling pathways that may play an important role in clinical outcome of stroke patients with low T3. In the present study, we obtained DEGs between T3-treated neuron and control from one microarray dataset in GEO. Subsequently, GO and Kyoto Encyclopedia of Genes and Genomes (KEGG) were performed followed by PPI network analysis on the basis of these DEGs. By using the bioinformatic method, further investigation on mechanism involved in clinical outcomes of low T3 syndrome was lighted, and it might provide some clues on potential biomarkers for clinical use and drug targets discovery.

## Materials and methods

### Study population

This study retrospectively enrolled 800 patients admitted to the department of neurology at the First Hospital Affiliated with Shandong First Medical University from December 2016 to June 2018 by random sampling. The Patients were diagnosed with AIS according to the American Heart Association/American Stroke Association guideline [14]. Four hundred twelve patients were recruited to AIS group according to inclusion criteria as follows: (1) age between 18 and 85 years; (2) stroke onset within 7 days before admission; (3) ischemic stroke confirmed by computerized tomography or magnetic resonance imaging at the time of admission. To avoid any confounding effects, 191 AIS patients were excluded according to the exclusion criteria including (a) no examination of diffusion-weighted image (DWI), inability to confirm the new characteristics of cerebral infarction (*n* = 78); (b) patients with haemorrhagic stroke, tumor, infection, inflammatory conditions, hematological diseases, and severe renal, liver or cardiopulmonary failure (*n* = 62); (c) patients with hyper- or hypothyroidism and treated with drugs known to interact with the thyroid gland (e.g. amiodarone, dopamine, β-blockers, corticosteroids, lithium, or phenytoin) (*n* = 51). Three hundred ten subjects at the age 18 to 85 years were recurtied to non-AIS group. One hundred twenty-eight subjects were excluded from non-AIS group based on the same exclusion criteria as described above: (a) *n* = 47, (b) *n* = 57 and (c) *n* = 24. Finally, 221 cases of AIS and 182 non-AIS control were included in our present study after inclusion and exclusion criteria were applied. All the patients had not been treated by thrombolysis or in the intensive care unit. The study protocol was approved by the ethics committee of Shandong Provincial Qianfoshan Hospital.

### Assessment of variables

Hypertension was defined as blood pressure levels ≥140/90 mmHg or by the use of anti- hypertensive drugs according to the diagnostic criteria of the European Society of Hypertension [15]. All patients were drew venous blood for routine laboratory after overnight fast within the first 24 h of the admission to hospital, including serum total cholesterol, triglyceride, low-density lipoprotein cholesterol and fasting plasma glucose, etc. The serum FT3, free thyroxine (FT4), and Thyroid Stimulating Hormone (TSH) levels were determined using the chemiluminometric assay (Cobas E610; Roche, Basel, Switzerland): the thyroid biochemistry intervals for referred laboratory were: FT3, 3.1–6.8 pmol/L; FT4, 12–22 pmol/L; and TSH, 0.27–4.0 mIU/L according to the American Thyroid Association criteria [16]. Diabetes was defined as a fasting blood glucose level ≥ 7.0 mmol/L, a random blood glucose level ≥ 11.0 mmol/L, or by the use of oral medication of glucose lowering drugs according to the diagnostic criteria of the American Diabetes Association [17]. Hyperlipidemia was defined as low density lipoprotein > 160 mg/dL, total cholesterol of > 240 mg/dL, triglycerides of > 150 mg/dL, or a history of oral medication with lipid-lowering drugs, which is in accordance with the diagnostic criteria of American College of Cardiology/American Heart Association [18]. Atrial fibrillation was diagnosed using electrocardiography upon admission and/or by the occurrence of paroxysmal atrial fibrillation during hospitalization. Stroke history was defined as a previous diagnosis of and treatment for stroke. Severe smoking was defined as smoking more than 6 months with ≥1 cigarette per day or daily smoking more than 20 cigarettes [19]. Excessive drinking is defined as drinking alcohol more than 25 g/day for adult males and more than 15 g/day for adult females according to Chinese Dietary Guidelines [20]. Stroke severity was gauged using the NIHSS on admission. The functional outcome was evaluated by the mRS at 3 months after stroke onset. The patients were classified into 2 outcome groups: poor (mRS > 2) and good (mRS ≤2) outcome. According to the subtyping criteria of the Trial of Org 10,172 in Acute Stroke Treatment (TOAST), the etiology of stroke was subtyped in each patient as follows: LAA, SVO, CE, stroke of other determined etiology (SOE),and stroke of undetermined etiology (SUE) [21].

### Imaging data

All of patients were subjected to a cerebral MRI examination with conventional MRI sequences (including sequenceT1, T2, T2 FLAIR, and DWI) within 72 h after official admission to clinical administration. Acute cerebral infarction was defined as a focal hyperintense lesion of ≥3 mm in diameter in the DWI. In this study, we addressed lacunar infarcts (≤15 mm in diameter) located in the subcortical white matter, basal ganglia, or thalamus. Periventricular white matter hyperintensity (PWMH) and subcortical white matter hyperintensity (SWMH) were evaluated based on their distinct subcortical distributions on the FLAIR image. Assessments of PWMH and SWMH were performed using the Fazekas rating scale (0–3, PWMH, 0–3, SWMH). Lacunes were defined as subcortical, fluid-filled (similar signal as cerebrospinal fluid) cavities (< 15 mm in diameter) thought to be present in the region of a previous acute small deep brain infarct or hemorrhage in the territory of a perforating arteriole. All the MRI findings were read and determined separately by an experienced neurologist and neuroradiologist who were blinded to the patients’ profiles.

### Microarray data

The gene expression profiles of GSE24793 were downloaded from GEO. The GSE24793 is performed on GPL1261, [Mouse430_2] Aymetrix Mouse Genome 430 2.0 Array. The GSE24793 data set contained 8 samples, including 6 h, 16 h, 24 h, 48 h and those untreated control.

### Data preprocessing and DEG screening

The downloaded platform and series of matrix files were converted using the R language software and annotation package. The ID corresponding to the probe name was converted into an international standard name for genes (gene symbol). Gene differential expression was performed using the limma package in R, with treated samples verse untreated ones. Multiple testing correction was done to control the overall error rate using the Benjamini-Hochberg false discovery rate (FDR). An FDR < 0.05 and a |log2 Fold Change (FC)| > 2 were used as the cut-off criterion to identify the final DEGs.

### Functional annotation and pathway analysis of DEGs

The DAVID database (https://david.ncifcrf.gov/) is an essential foundation for the success of any high-throughput gene function analysis. The functional and pathway enrichment of the proteins encoded by candidate genes were analyzed, and these genes were annotated using the DAVID database. GO annotations and KEGG pathways were performed using a DAVID online tool on the screened DEGs. In this study, we analyzed the DEGs that were significantly up- and downregulated as determined from those microarray data, and a *P*-value of < 0.05 was considered statistically significant.

### Analysis of protein interaction networks

The online Search Tool for the Retrieval of Interacting Genes (STRING) database was used to construct PPI network of the DEGs. An interaction score of > 0.4 (medium confidence score) was considered significant and the PPI was visualized. Subsequently, the hub genes were selected according to connection degree by Cytoscape software. Moreover, Molecular Complex Detection (MCODE) was applied to find clusters of genes in the PPI network. ‘degree cutoff = 2’, ‘node score cutoff = 0.2’, ‘k-core = 2’ and ‘max. depth = 100’ were set as the cut-off criteria.

### Statistical analysis

Statistical analyses were conducted using the Statistical Package for the Social Sciences (SPSS 20.0) for windows (SPSS Inc., Chicago, IL) and GraphPad Prism 6 software package (GraphPad software, SanDiego, CA). Continuous data were expressed as mean ± standard deviation (SD) or median (interquartile range). Differences in continuous variables between 2 groups were determined by Student’s *t* test. Non-normally distributed data were compared using Mann-Whitney U test and Kruskal-Wallis test. Noncontinuous and categorical data were compared with the chi-square test or Fisher exact test. To adjust for the traditional risk factors, multivariable logistic regression analysis was performed by binary logistic regression analysis, which allows adjust for confounding factors. One-way analysis of variance was carried out to analyze the difference in the serum FT3 levels for each TOAST subtype. A receiver-operating characteristic curve was used to determine the sensitivity and specificity of FT3. Optimal cut-off value of FT3 in the receiver-operating characteristic curve was determined at the level with the highest Youden index (sensitivity+specificity-1). The accuracy of the test was assessed measuring the area under the ROC curve (AUROC). A *P* value of less than 0.05 indicates statistical significance.

## Results

### The demographic and baseline characteristics

The demographic and baseline characteristics are shown in Table 1. According to univariate analysis exploring each variable separately in these data, the number of patients diagnosed as hypertension (*P* = 0.003), diabetes mellitus (*P* = 0.001), hyperlipidemia (*P* = 0.011) and hyperhomocysteinemia (*P* < 0.0001) in AIS group was significantly higher than those in non-AIS group. In addition, there existed significant differences in atrial fibrillation (*P* = 0.022), smoking (*P* < 0.001), drinking (*P* < 0.0001), stroke history (*P* < 0.0001), cerebral small vessel-related lesions [PWMH (*P* = 0.006), SWMH (*P* = 0.012) and lacuna (*P* < 0.0001) between AIS group and non-AIS control. While recruited subjects in AIS group had lower FT3 levels (*P* = 0 .002) and the ratio of FT3 to FT4 (*P* = 0 .004) compared with patients without AIS, there were no significant differences in the FT4 and TSH levels between them.

**Table 1:** Demographic and baseline characteristics

Variables	Total	AIS	Non-AIS	*p* value
Sex(n)	403	221	182	NS
Male	233	129	104	
Female	170	92	78	
Age (years)	64.65 ± 10.85	66.80 ± 7.78	63.13 ± 12.51	NS
Hypertension (*n*)				0.003
Yes	165	105	60	
No	238	116	122	
Diabetes (n)				0.001
Yes	116	79	37	
No	287	142	145	
Hyperlipidemia (n)				0.011
Yes	161	101	60	
No	242	120	122	
Hyperhomocysteinemia (n)				< 0.0001
Yes (> 15 μmol/L)	111	85	26	
No (≦ 15 μmol/L)	292	136	156	
Smoking (n)				< 0.0001
Yes	128	88	40	
No	275	133	142	
Drinking (n)				< 0.0001
Yes	94	70	24	
No	309	151	158	
Atrial fibrillation (n)				0.022
Yes	43	31	12	
No	360	190	170	
Stroke history (n)				< 0.0001
Yes	109	89	20	
No	281	132	149	
with Periventricular WMH				0.006
Grade 1	81	46	35	
Grade 2	44	32	11	
Grade 3	26	23	3	
with subcortical WMH				0.012
Grade 1	74	42	32	
Grade 2	43	31	12	
Grade 3	29	25	4	
with lacunas (n)				< 0.0001
Yes	163	113	50	
No	240	108	132	
FT3, pmol/L	4.5870 ± 0.7737	4.26 ± 0.49	4.65 ± 0.24	0.002
FT4, pmol/L	16.59 ± 2.42	16.77 ± 2.52	16.35 ± 2.57	NS
TSH, uIU/ml	2.33 ± 0.74	1.95 ± 0.54	2.49 ± 0.65	NS
FT3/FT4	0.27 ± 0.05	0.25 ± 0.04	0.29 ± 0.05	0.004

*AIS* acute ischemic stroke; *WMH* white matter hyperintensity; *FT3* free triiodothyronine; *FT4* free thyroxine; *TSH* thyroid stimulating hormone

### The relationship between serum level of FT3 and clinical factors of AIS patients

**Fig. 1:**
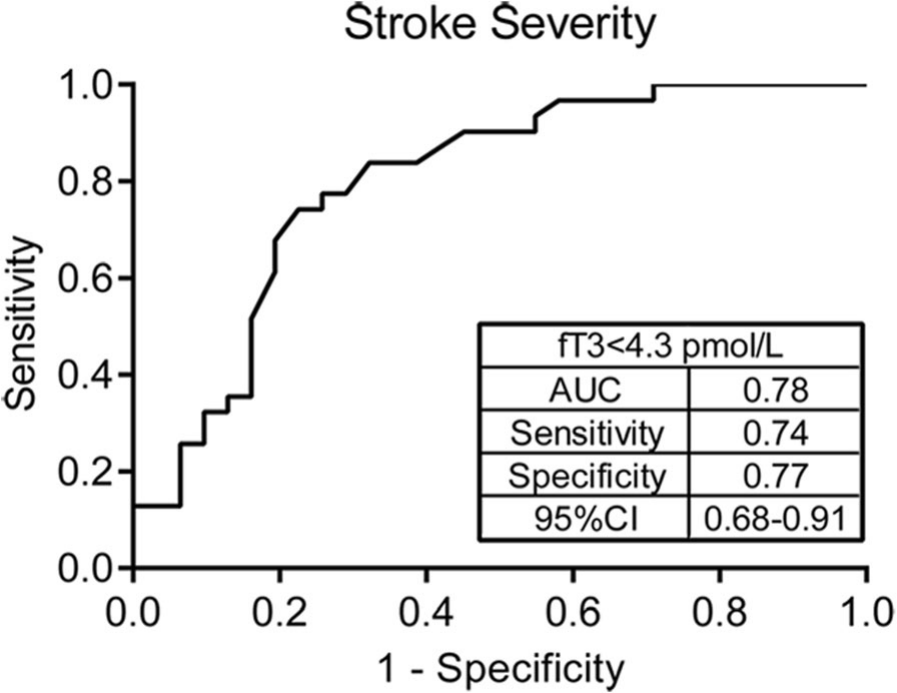
Receiver-operating characteristic analysis (ROC) of FT3 for the severity of ischemic stroke. AUC = area under the curve

On analysis of association between FT3 and stroke risky factors, it was found that the lower level of FT3 was more likely to be detected in the AIS patients with NIHSS on admission > 3 (*P* = 0.013) and 3-month mRS > 2 (*P* = 0.002). There were no significant association between FT3 level and other factors including TIA history, recurrence, hypertension, diabetes, hyperlipidemia, hyperhomocysteinemia, smoke, drinking, atrial fibrillation, stroke history and cerebral small vessel-related lesions (Table 2). In addition, the ROC curve analysis of FT3 predicting stroke severity on admission (NIHSS score) indicated that the cut-off value of FT3 was 4.30 pmol/L with sensitivity of 74%, specificity of 77% and area under the curve (AUC) of 0.78 (Fig. 1).

**Table 2:** Serum level of FT3 and clinical characteristics in AIS patients

Clinical features	FT3 (pmol/L)	*p* value
TIA history		
Yes	4.62 ± 0.79	NS
No	4.27 ± 0.67	
Recurrence		
Yes	4.68 ± 0.51	NS
No	4.47 ± 0.67	
Hypertension		
Yes	4.43 ± 0.62	NS
No	4.67 ± 0.48	
Diabetes		
Yes	4.84 ± 0.51	NS
No	4.43 ± 0.62	
Hyperlipidemia		
Yes	4.65 ± 0.42	NS
No	4.57 ± 0.38	
Hyperhomocysteinemia		
Yes (>15 μmol/L)	4.54 ± 0.63	NS
No (≤15 μmol/L)	4.73 ± 0.53	
Smoking (n)		
Yes	4.74 ± 0.57	NS
No	4.48 ± 0.55	
Drinking (n)		
Yes	4.93 ± 0.41	NS
No	4.42 ± 0.54	
Atrial fibrillation		
Yes	4.98 ± 0.43	NS
No	4.63 ± 0.53	
Stroke history		
Yes	4.86 ± 0.41	NS
No	4.56 ± 0.51	
with periventricular WMH		
Grade 1	4.43 ± 0.57	NS
Grade 2	4.17 ± 0.52	
Grade 3	4.15 ± 0.49	
with subcortical WMH		
Grade 1	4.45 ± 0.54	NS
Grade 2	4.21 ± 0.46	
Grade 3	4.13 ± 0.0.56	
with lacunes		
Yes	4.24 ± 0.55	NS
No	4.66 ± 0.62	
NIHSS on admission		
> 3	4.11 ± 0.52	0.013
≤ 3	4.87 ± 0.24	
mRS (3 mos)		
> 2	4.17 ± 0.35	0.002
≤ 2	4.97 ± 0.32	

*AIS* acute ischemic stroke; *TIA* transient ischemic attack; *WMH* white matterhyperintensities; *NIHSS* National Institutes of Health Stroke Scale; *mRS* modified Rankin Scale

### Comparison of serum level of FT3 in the different stroke subtypes according to TOAST

**Fig. 2:**
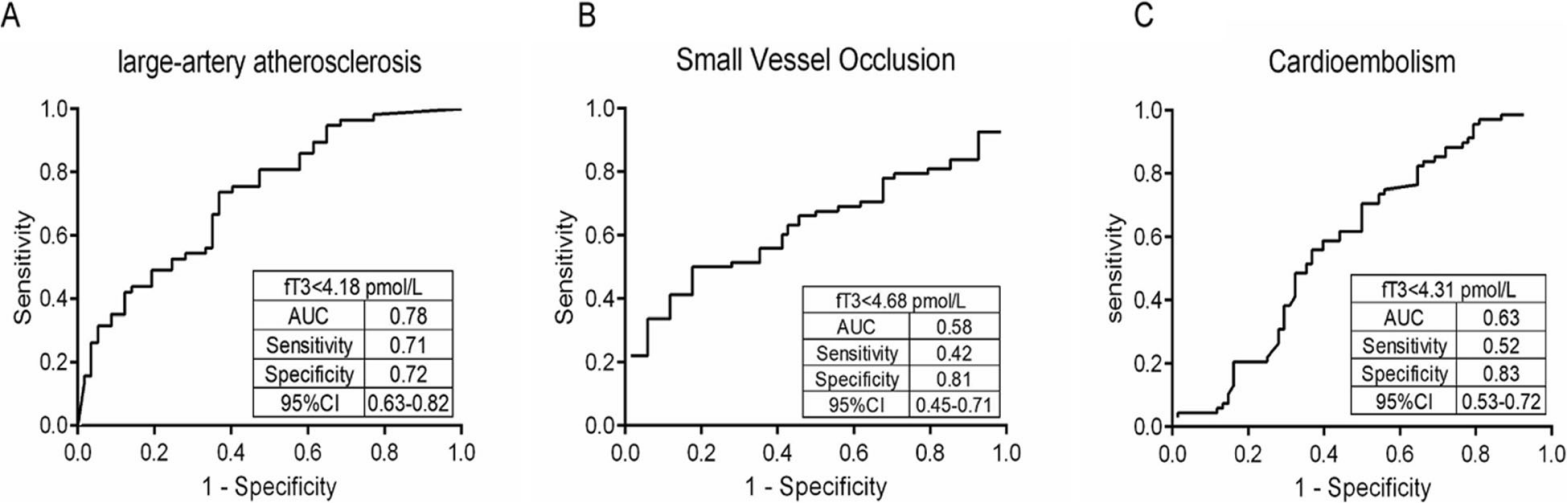
Receiver-operating characteristic analysis (ROC) of FT3 for 3 subtypes of ischemic stroke. AUC = area under the curve

As shown at Table 3, it was found that significant difference of level of FT3 existed among the stroke subtypes (*F* = 20.120, *P* < 0.001), and FT3 level in LAA subtype and CE subtype was moderately lower that in SVO subtype. In Table 4, it was indicated that the patients with LAA and CE had less FT3 value compared with those in SVO (*P* < 0.001), whereas no significant differences between LAA and CE were found (*P* = 0.685). ROC curves for FT3 distinguishing 3 subtypes of ischemic stroke are shown in Fig. 2. The best predictive value for FT3 was found in LAA group, in which the cut-off value of FT3 was 4.18 pmol/L (sensitivity 71%; specificity 72%; AUC 0.78). In CE group, it was 4.31 pmol/L (sensitivity 52%; specificity 83%; AUC 0.63). In SVO group, it was 4.68 pmol/L (sensitivity 42%; specificity 81%; AUC 0.58).

**Table 3:** Comparison of serum FT3 levels in different clinical subtype of AIS

	LAA	CE	SVO	*F* value	*P* value
FT3 (pmol/L)	4.14 ± 0.41	4.21 ± 0.40	4.87 ± 0.36	20.120	< 0.001

*FT3* free triiodothyronine; *AIS* acute ischemic stroke; *LAA* large-artery atherosclerosis; *CE* cardioembolism; *SVO* small-vessel occlusion

**Table 4:** Results of the LSD test for serum FT3 levels in each group

Group	Mean difference	SE	95% confidence interval	*P* value
LAA				
CE	−0.05	0.11	(−0.28) - (0.18)	0.685
SVO	−0.63	0.12	(−0.86) - (−0.39)	< 0.001
SVO				
CE	0.58	0.15	0.35–0.81	< 0.001

*FT3* free triiodothyronine; *LSD* least significant difference; *LAA* large-artery atherosclerosis; *CE* cardioembolism; *SVO* small-vessel occlusion

### Unadjusted association of FT3, FT4 and TSH with mRS at 3 months after stroke

The unadjusted comparison of FT3, FT4 and TSH level in clinical outcomes after AIS is presented in Table 5. We found that AIS patients who presented as worsen prognosis (mRS > 2 at 3 months after stroke) showed lower FT3 level compared with those with 3-month mRS ≤ 2 (4.17 ± 0.35 vs 4.97 ± 0.32, *P* = 0.002), while there were no significant differences in FT4 (*P* = 0.446) and TSH (*P* = 0.548) level for clinical prognosis, which suggested that FT3 had potential function for prognostic prediction of stroke.

**Table 5:** Unadjusted comparison of FT3, FT4 and TSH in clinical outcomes

	Worsen outcomes (3 months)		
	Present	Absent	*P*
FT3, pmol/L	4.17 ± 0.35	4.97 ± 0.32	0.002
FT4, pmol/L	16.02 ± 1.84	16.71 ± 1.53	0.446
TSH, uIU/ml	2.15 ± 0.42	2.50 ± 0.84	0.548

*FT3* free triiodothyronine; *FT4* free thyroxine; *TSH* thyroid stimulating hormone

### Relationship between FT3 and clinical outcome

**Fig. 3:**
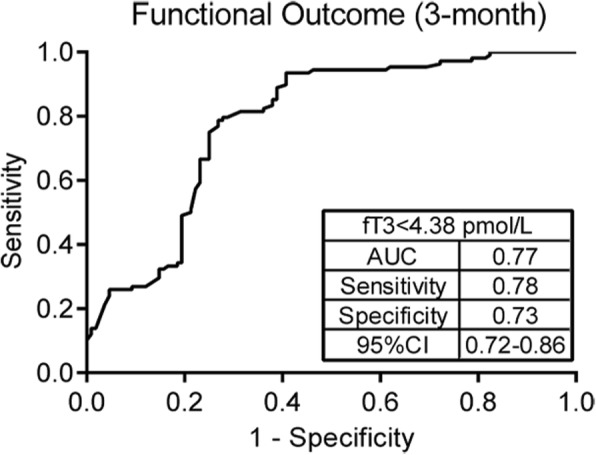
Receiver-operating characteristic analysis (ROC) of FT3 for the prediction of 3-month neurological function after stroke

As presented in Table 6, univariate logistic regression analyses revealed that adverse clinical outcome was significantly associated with lower FT3 concentration (OR 0.370, 95%CI 0.22–0.54, *P =* 0.013), higher NIHSS score (OR 1.742, 95%CI 1.12–2.34, *P =* 0.008) and age (OR 1.251, 95%CI 1.01–1.71, *P =* 0.031). Furthermore, binary logistic regression analyses revealed that poor outcome was associated with lower FT3 level on admission (OR 0.348, 95%CI 0.18–0.72, *P* = 0.007). In addition, age (OR 1.471, 95%CI 1.22–1.78, *P* = 0.015) and NIHSS on admission (OR 1.410, 95%CI 1.17–1.85; *P* < 0.001) also served as significant outcome predictors. Conversely, some other risky factors, including hypertension, diabetes, hyperlipidemia, etc., were barely considered to be remarkably predictive factors of poor neurological prognosis (all *P* > 0.05). Accordingly, our data drawn a preliminary conclusion that FT3 emerged as an independent and significant predictor for 3-month neurological outcome. Additionally, the ROC curve analysis (Fig. 3) revealed that serum level of FT3 < 4.38 pmol/L in AIS patients was a powerful predictor of neurological outcomes (sensitivity 78%; specificity 73%; AUC 0.77).

**Table 6:** Multiple logistic regression analysis of poor functional outcome predictors

Variables	OR	95% CI	*p*-value
Univariate analysis			
Sex(n)	0.440	0.25–3.97	0.468
Age (years)	1.251	1.01–1.71	0.031
Hypertension (*n*)	0.922	0.58–1.72	0.609
Diabetes (n)	1.780	1.14–2.85	0.064
Hyperlipidemia (n)	1.240	0.51–1.73	0.750
Hyperhomocysteinemia (n)	1.511	0.72–1.86	0.355
Smoking (n)	1.753	1.05–2.96	0.450
Drinking (n)	1.690	1.24–2.19	0.238
Atrial fibrillation (n)	1.313	0.61–2.42	0.403
Stroke history (n)	0.793	0.51–1.54	0.833
with periventricular WMH	1.597	1.04–4.37	0.369
with subcortical WMH	1.415	1.02–2.37	0.457
with lacunes(n)	1.263	0.75–2.15	0.326
FT3, pmol/L	0.370	0.22–0.54	0.013
FT4, pmol/L	1.027	0.92–1.12	0.573
TSH, uIU/ml	0.836	0.62–1.08	0.201
FT3/FT4	0.001	0.00–0.01	0.001
NIHSS on admission	1.742	1.12–2.34	0.008
Multivariate analysis			
Age (years)	1.471	1.22–1.78	0.015
FT3, pmol/L	0.348	0.18–0.72	0.007
NIHSS on admission	1.410	1.17–1.85	< 0.001

*WMH* white matter hyperintensity; *FT3* free triiodothyronine; *FT4* free thyroxine; *TSH* thyroid stimulating hormone; *NIHSS* National Institutes of Health Stroke Scale

### Data preprocessing and identification of DEGs

**Fig. 4:**
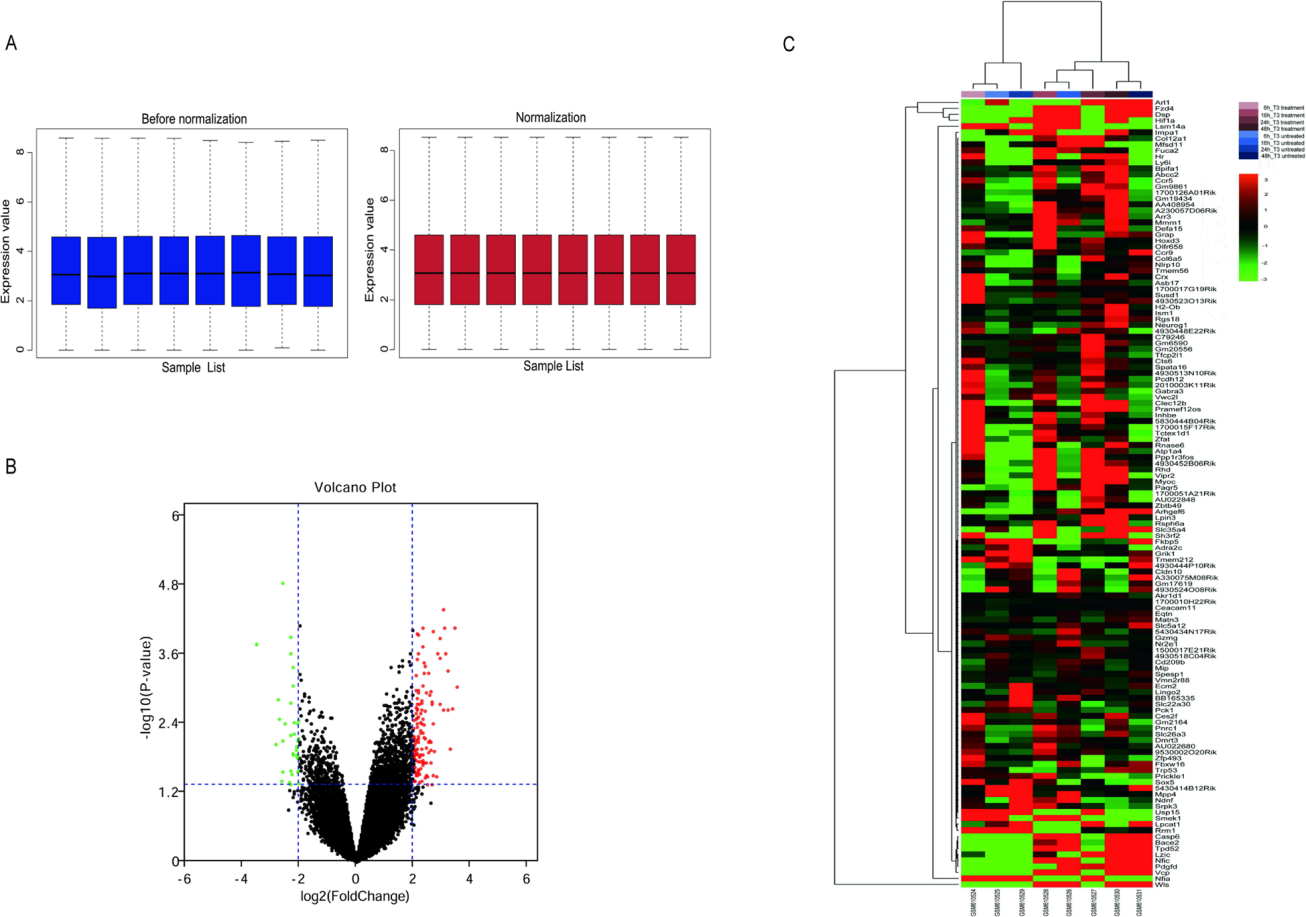
Differences in gene expression profiles between neuron treated by T3 and sham groups. **a**. Standardization of GSE data. The blue bar represents the data before normalization, and the red bar represents the normalized data. Black lines in the boxes represent medians. **b**. Volcano plots show the distributions of gene expression between the treated and sham groups. The horizontal line corresponds to a 2-fold change up or down, and the vertical line represents a *P*-value< 0.05. The red points represent upregulated genes on the basis of |fold change| > 2.0 and *P* value < 0.05. The green points represent downregulated genes screened on the basis of |fold change| > 2.0 and a corrected *P*-value < 0.05. The black points represent genes with no significant difference. FC, fold change. **c**. Hierarchical clustering heatmap of DEGs screened on the basis of |fold change| > 2.0 and a corrected *P*-value < 0.05.The left vertical axis shows clusters of DEGs, and the above horizontal axis shows clusters of samples. Red represents up-regulated genes and green represents down-regulated genes, and black indicates no significant changes in gene expression; gray indicates that the signal strength of genes was not high enough to be detected. DEGs, differentially expressed genes

The expressive distribution characteristics of the dataset GSE24793 prior to and following data pretreatment are shown in Fig. 4a. Their median values were almost on a straight line, indicating that the raw data were normalized successfully. When the GSE24793 dataset was screened by the limma package (corrected *P*-value < 0.05, logFC > 2), 146 DEGs were obtained. Among them, 33 downregulated genes and 113 upregulated genes were identified and shown in Fig. 4b. The cluster heatmap of these DEGs is shown in Fig. 4c.

### Function and pathway enrichment analysis

**Fig. 5:**
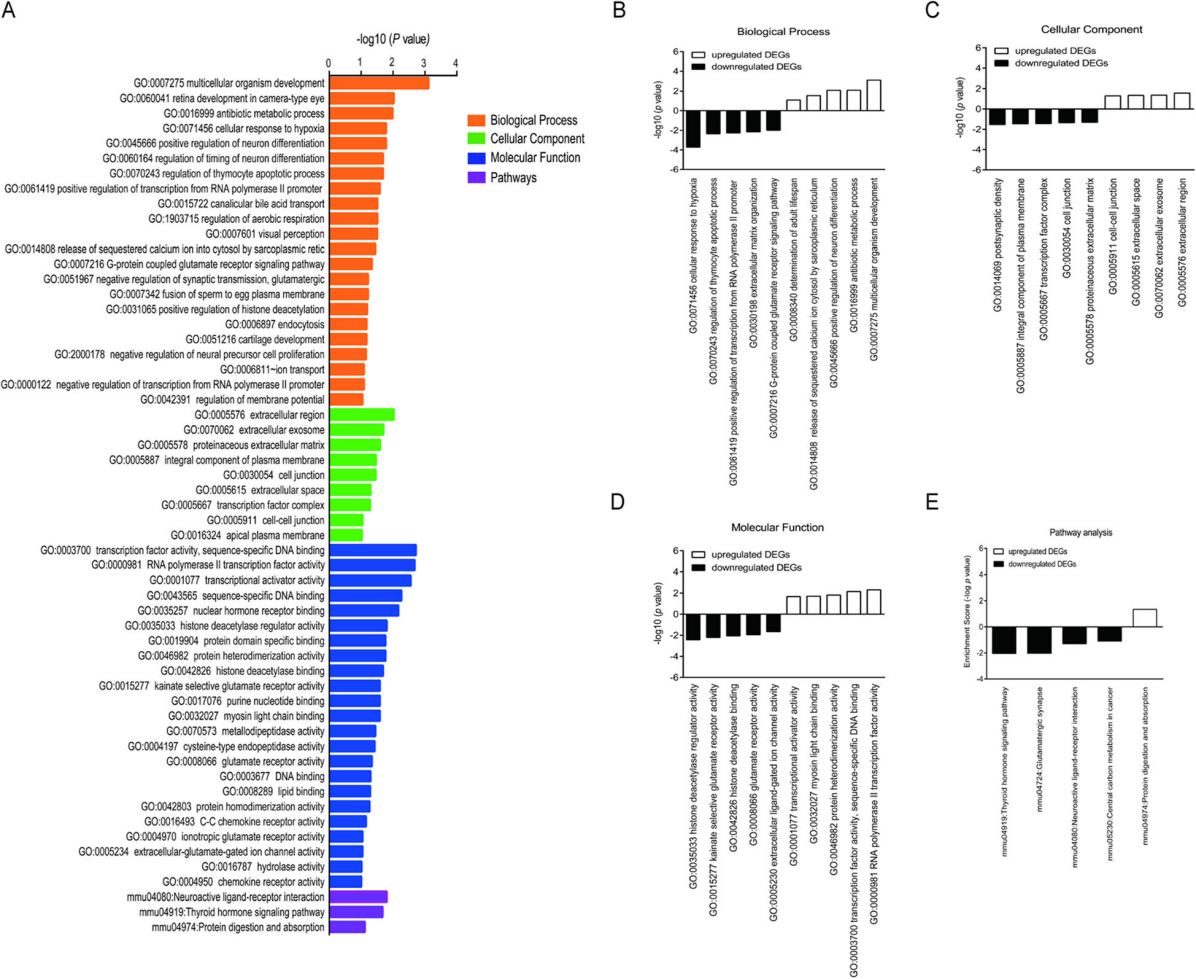
GO and KEGG enrichment analysis of DEGs in Purkinje cells treated by T3 and sham control. **a**. GO and KEGG enrichment significance items of DEGs in different functional groups. The horizontal axis represents the enrichment score of DEGs. The vertical axis represents the GO categories. **b**. The top 5 significantly downregulated and upregulated BP enrichment from DEGs. The horizontal axis represents the BP items. The vertical axis represents the enrichment score of DEGs. **c**. The top 5 significantly dwnregulated and the 4 upregulated CC enrichment from DEGs. The horizontal axis represents the CC items. The vertical axis represents the enrichment score of DEGs. **d**. The top 5 significantly downregulated and upregulated MF enrichment from DEGs. The horizontal axis represents the MF items. The vertical axis represents the enrichment score of DEGs. **e**. The downregulated and upregulated KEGG pathways analysis from DEGs. The horizontal axis represents the KEGG items. The vertical axis represents the enrichment score of DEGs. GO, gene ontology; KEGG, Kyoto Encyclopedia of Genes and Genomes; DEGs, differentially expressed genes; BP, biological process; CC, cellular component; MF, molecular function

Biological annotation of 146 DEGs was performed using the DAVID online analysis tool, and GO functional enrichments and KEGG pathway of up-and downregulated genes with a *P*-value of 0.05 were obtained (Fig. 5a). GO analysis of DEGs was divided into three functional groups, including biological processes (BP), molecular function (MF) and cell composition (CC). As for BP group, the top 5 BP of the upregulated genes were mainly involved in multicellular organism development, antibiotic metabolic process, positive regulation of neuron differentiation, release of sequestered calcium ion into cytosol by sarcoplasm icreticulum and determination of adult lifespan while the top 5 BP derived from the downregulated genes were concentrated in cellular response to hypoxia, regulation of thymocyte apoptotic process, positive regulation of transcription from RNA polymerase II promoter in response to hypoxia, extracellular matrix organization and G-protein coupled glutamate receptor signaling pathway (Fig. 5b). In the MF group, the top 5 MF from upregulated genes were mainly enriched in RNA polymerase II transcription factor activity, transcription factor activity, protein heterodimerization activity, myosin light chainbinding and transcriptional activator activity, RNA polymerase II corepromoter proximalregion sequence-specific binding (Fig. 5c), whereas the downregulated genes were mainly enriched in histone deacetylase regulator activity, kainate selective glutamate receptor activity, histone deacetylase binding, glutamate receptor activity and extra cellular-glutamate-gated ion channel activity (Fig. 5c). In the CC group, the upregulated genes were mainly enriched into extracellular region, extracellular exosome, extracellular space and cell-cell junction (Fig. 5d). The top 5 CC from downregulated genes were enriched in construction of postsynaptic density, integral component of plasma membrane, transcription factorcomplex, cell junction and proteinaceous extracellularmatrix (Fig. 5d). KEGG pathways enrichment analysis indicated that the upregulated genes were enriched into protein digestion and absorption (Fig. 5e). The downregulated genes were enriched in thyroidhormone signaling pathway, glutamatergic synapse, neuroactive ligand-receptor interaction and central carbon metabolism in cancer (Fig. 5e).

### PPI network construction and module analysis

**Fig. 6:**
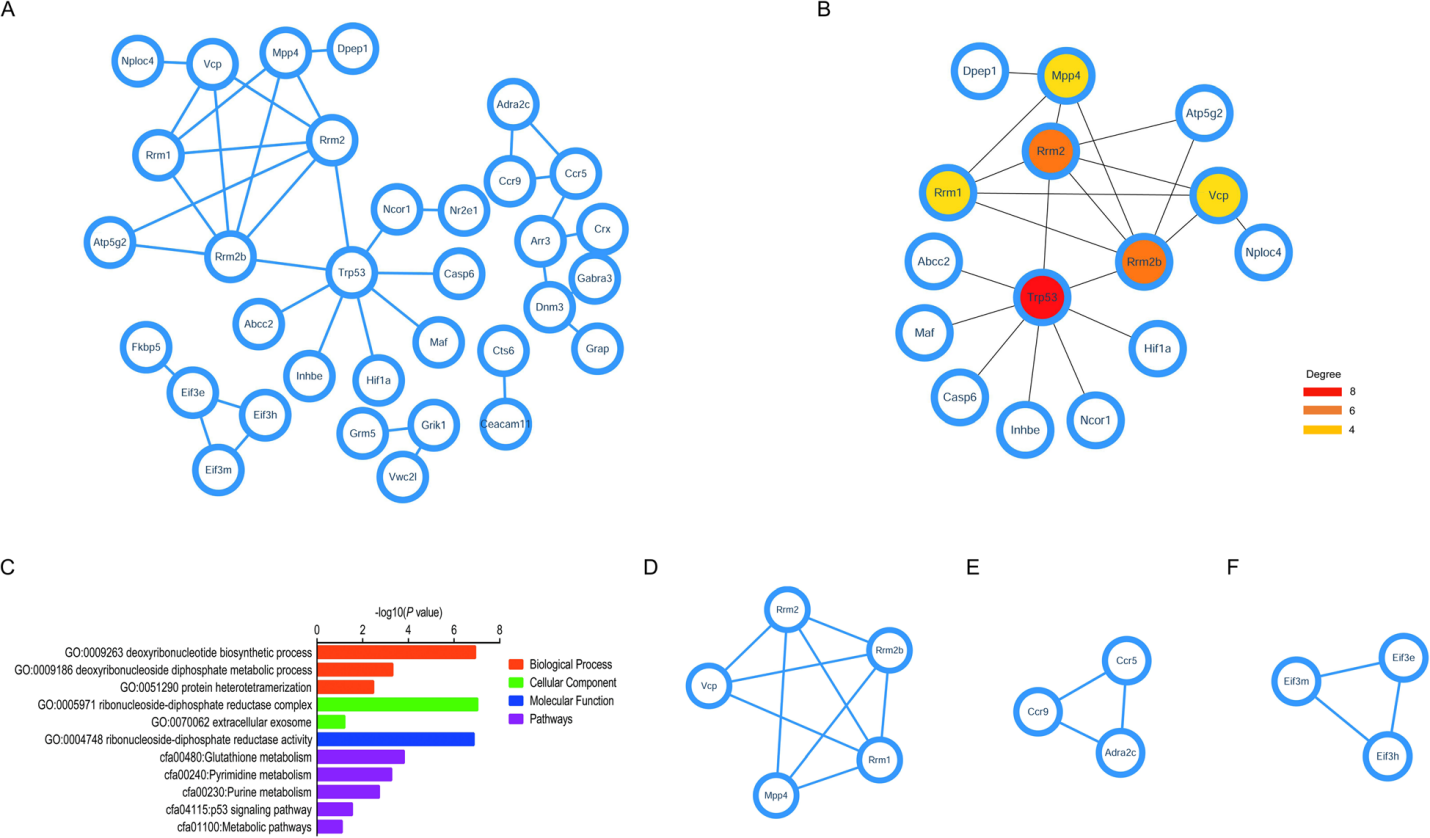
The protein-protein interaction (PPI) network, the most significant module and functional analysis of DEGs. **a** and **b**. The PPI network of DEGs and six hub genes constructed by Cytoscape. **c**. GO and KEGG pathway analysis of hub genes. **d**, **e** and **f**. The modules obtained from PPI network. The nodes represented the genes, and the edges represented the corresponding PPI pairs. The color depth and size of nodes refer to the ‘degree’ calculated by Cytoscape. The size of edges refers to the combined-score calculated by Cytoscape. Low value of degree is homologous to small size and bright color of nodes. Small size of edges stands for low value of combined-score

PPI networks were constructed on the basis of the STRING database. After removing the isolated and partially connected nodes, a complex network of DEGs is displayed in Fig. 6a. When ‘Degree ≥4’ was set as the cut-off criterion, a total of 6 genes were selected as hub genes from DEGs, and the full names, abbreviations and functions for these hub genes are shown in Table 7. It was found that the biological functions of 6 hub genes are mainly referred to cell cycle, DNA repair and DNA synthesis. The functional annotation of these hub genes is shown in Fig. 6b. MCODE analysis of PPI networks was indicative of a total of 3 modules. Module A, the most significant module, included 5 nodes (MMP4, RRM2, RRM2B, RRM1 and VCP) and 9 edges, of which MPP4 was identified as the seed gene (Fig. 6c). Module B represented 3 nodes (ADRA2C, CCR9 and CCR5) and 3 edges of which ADRA2C was considered as the seed gene (Fig. 6d). Module C contained 3 nodes (EIF3E, EIF3M and EIF3H) and 3 edges, and EIF3E was the seed gene (Fig. 6e).

**Table 7:** Functional roles of hub genes

NO.	Gene symbol	Full name	Function
1	TRP53	Tumor protein P53	TRP53 is a transcription factor which can regulate cell cycle arrest, apoptosis, senescence, DNA repair, or changes in metabolism
2	RRM2	Ribonucleotide reductase regulatory subunit M2	RRM2 is involved in catalyzing the formation of deoxyribonucleotides from ribonucleotides.
3	RRM2B	Ribonucleotide reductase regulatory TP53 inducible subunit M2B	RRM2B encodes the small subunit of a p53-inducible ribonucleotide reductase which is necessary for DNA synthesis.
4	RRM1	Ribonucleotide reductase catalytic subunit M1	RRM1 has been implicated in DNA replication as well as multiple DNA repair processes
5	VCP	Valosin containing protein	VCP is involved in protein degradation, intracellular membrane fusion, DNA repair and replication and activation of the NF-kappa B pathway
6	MPP4	Membrane palmitoylated protein 4	MPP4 is associated with photoreceptor polarity and the organization of specialized intercellular junctions

## Discussion

T3 has an ability to induce hypothermia, anti-edema, anti-apoptotic, anti-inflammation and vasodilatation activates [22, 23]. Specially, FT3, the bioactive form of T3, also plays an important role in neurogenesis in the all stages of brain development [24]. Our results have indicated that the lower FT3 in patients with AIS was associated with greater baseline severity of ischemic stroke on admission (as defined by the NIHSS scores) and worse prognosis (as defined by the mRS). Additionally, it may be more likely to occur in patients with LAA and CE subtypes. In order to illustrate the possible mechanism underlying clinical outcome with low FT3, a bioinformatics analysis was performed to show that the worsen prognosis of AIS patients was associated with the disregulation of neurogenesis low FT3 induced, including multicellular organism development, extracellular matrix organization, glutamatergic synapse, etc.

Previous studies have reported that serum concentrations of FT3 are altered in the acute phase of stroke [5, 25], which are consistent with our study. It should be noted that while there exists an association between alteration of serum FT3 and stroke onset, it implies no causation. In fact, low FT3 is a phenomenon observed in several acute conditions, not only following stroke. It has been proposed that the acute development of low FT3 levels following severe illnesses, including patients with acute cardiac events; after brain tumor surgery and patients with respiratory failure [26–28], occurs due to the induction of decreased peripheral conversion of FT4 to FT3 and aberrant thyroid hormone metabolism in CNS [29, 30]. These changes may occur for various reasons, including damage to the hypothalamic-pituitary axis (HPA) following AIS, accumulation of proinflammatory cytokines as well as metabolic changes related to free fatty acid and bilirubin levels [31–33]. It was reported that the increased levels of IL-6 may lead to increased CRP as well as inhibition of the HPA, which subsequently depresses thyroid stimulation [34]. Thus, the acute stress following stroke may lead to the low FT3 levels versus low FT3 causing occurrence of stroke [5].

As for the diversified subtypes of AIS, our data showed that the level of serum FT3 in LAA and CE was significantly lower than that in the SVO group, and yet no significant difference was found between LAA and CE. Similar findings have been reported by Guan Wu et al., who found that low FT3 level is prone to occur in patients with LAA cerebral infarction [35]. Of interest, our study revealed that most of patients within LAA and CE had more severe neurological deficits compared with those in SVO. Several studies also collectively addressed that low FT3, in the setting of a normal TSH level, was correlated with worse stroke severity upon presentation [5, 25, 36]. Thus, it may suggest that the stimulus of acute neurological impairment either directly or indirectly triggered the occurrence of lower serum FT3, but the precise mechanism through our speculation remains to be unveiled. A previous study pointed out higher expression of hsCRP, IL-6 and ferritin in serum of LAA and CE patients [37], which may be one of factors that contributed to the decrease of serum FT3.

Low FT3 level on admission was also reported to be an important predictor for neurological disability valued by 3-month mRS score [25, 34]. These results were consistent with the results of our study that the patients with lower T3 represented higher baseline stroke severity and worse functional outcome. However, some other studies revealed that subclinical hypothyroidism may in fact be a crucial factor of better prognosis following stroke [9, 10]. These studies depicted the associations between stroke outcome and TSH (not T3) levels, but the patients involved in these studies were not overtly hypothyroid, in fact they had the value of serum T3 within the reference range [9]. Interestingly, the patients with elevated TSH in this study who did better than those with normal TSH also had higher average T3 levels [9]. Therefore, even though the researchers divided patients based on TSH status and not serum T3 levels, their results were also in accord with the results of our study.

The precise mechanisms underlying low FT3 acting as a predictor of worsen prognosis remain to fully established. T3 is especially required for the generation and maturation of neurons and axonal myelination partly owing to its higher affinity for thyroid receptors (TRs) than T4 which is more abundant [38, 39]. Several studies documented neurotropic actions of T3 that can be potentially neuroprotective under stroke, including reduction of developmental neuronal apoptosis, decreased glutamate transfer into brain cell and increased brain-derived neurotrophic factor (BDNF) [40–43]. In the one side, microarray technology and bioinformatic analysis have been widely used to screen genetic alterations at the genome level. To the best of our knowledge, there was no report that provided the gene expression profile of cerebral neuron w/wo stimulus of T3 for 3 months. Therefore, instead we analyzed gene expression changes of Purkinje cells exposed to T3 that, to some extent, uncover molecular mechanisms through which low FT3 on admission was indicative of worsen prognosis after 3 months. As indicated by bioinformatic analysis, BP analysis of upregulated genes were mainly enriched in multicellular organism development and positive regulation of neuron differentiation. Recent study pointed that the periinfarct zone mimics early stages of development in the brain with increased plasticity and a permissive microenvironment for remodeling [44]. Hence, it was supposed that low serum FT3 may act as an anti-agonist of neuron development and plasticity in the periinfarct against clinical recover of AIS patients. Besides, BP of downregulated genes were mainly attributed to cellular response to hypoxia, positive regulation of transcription in response to hypoxia and extracellular matrix organization. It was reported that earlier T3-treatment could promote the expression of hypoxia-mediated genes (VEGF, HIF2α, c-Jun) and KLF9, which may trigger cellular growth that is important for FT3-mediated neuronal protection and recovery [23]. Also, FT3 was reported to alter the production and organization of the extracellular matrix (ECM) proteins and proteoglycans, producing a high-quality substrate for neuronal differentiation [45]. In addition, the most significant KEGG pathway was enriched into neuroactive ligand-receptor interaction in which the DEGs consisted of GRM5, GRIK1, GRIK3, GABRA3, ADRA2C and VIPR2. As for GRM5, Its activation in astrocyte endfeet has been reported to worsen brain edema in a rat model of transient middle cerebral artery occlusion [46]. Both GRIK1 and GRIK3, as members of the glutamate kainate receptor family, play a critical role in synaptic potentiation important for neurogenesis and neurotransmission [47]. GABRA3, as a regulator of excitatory and inhibitory neurochemical expressions, has an ability of mediating development of the subcortical cerebellar structures [48]. ADRA2C dysregulation during embryonic development has an inhibitory capacity of the migration of cortical Interneurons involved in the formation of cortical circuits [49]. VIPR2 is responsible for reduction in proinflammatory cytokine release and increase in GM-CSF transcripts in CD4(+) T cells, and the use of VIPR2-selective agonists as neuroprotective agents is beneficial to PD treatment [50]. Thus, a deep understanding of these pathways and related molecules may provide us with the potential targets for prevention and therapy of stroke with low FT3, and further experiments are required to validate their function in stroke.

In addition, the PPI network provided an overview of DEGs functional connections, in which six hub genes with degrees ≥4 were selected. TRP53, as one of hub genes, was found to be upregulated in the ischemic region in neuronal cells of WT mice, and plays a significant role in hippocampal neuron death through autophagy and apoptosis following cerebral ischemia-reperfusion [51]. However, literature retrieval results showed little evidences that other hub genes MPP4, RRM2, RRM2B, RRM1 and VCP contributed to the molecular mechanism of stroke prognosis. Interestingly, module analysis of the PPI network revealed that these five genes above were also enriched into the same module, suggesting that their biological functions were analogical to some extent. KEGG analysis for these hub genes disclosed glutathione metabolism, pyrimidine metabolism, purine metabolism, p53 signaling pathways and metabolic pathways involved, indicating these genes were responsible for DNA synthesis, DNA repair, apoptosis and autophagy of neuron. Accordingly, we speculate that these hub genes may act as potential candidates for molecular mechanism underlying stroke prognosis, and further research is needed to confirm our hypothesis.

However, there are some limitations to our study. Our study merely recruited Han Chinese patients from the central and northern parts of China. The constitute of recruited patients, therefore, was unable to represent all AIS patients. In addition, there existed no more than 3-months follow-up in our study. Hence, multicenter clinical observation trials with larger sample sizes are needed to clarify whether measurement of FT3 helps predict the clinical prognosis of AIS. Another limitation showed that no gene expression profile was found to present the dynamic change of pathogenic genes in cerebral neuron exposed to FT3, and hence the genetic changes of Purkinje cells exposed to FT3 within 48 h could only give us some clues on molecular mechanism through which low FT3 could retard recovery of AIS patients, and further investigation is required to confirm the biological function of the identified genes in our study.

## In conclusion

Our study showed that the lower FT3 value was not only associated with great severity of AIS, but it serves as a predictor of 3-month worsen neurological outcomes after AIS. To get access to the molecular mechanism underlying the clinical prognosis, a comprehensive bioinformatics analysis was performed to reveal a range of genes which are involved in neuron development, plasticity and neurite outgrowth, and potentially serve as the promising candidates for alleviating neurological outcomes lower FT3 induces. Those identified genes and signaling pathways drive us for further clinical investigation and application.

## Data Availability

The datasets used and/or analysed during the current study are available from the corresponding author on reasonable request.

## Abbreviations

AIS: Acute ischemic stroke
AQP4: Aquaporin 4
BNDF: Brain-derived neurotrophic factor
BP: Biological processes
CC: Cell composition
CE: Cardioembolism
DEGs: Differentially expressed genes
ECM: Extracellular matrix
FC: Fold Change
FDR: False discovery rate
FT3: Free triiodothyronine
FT4: Free thyroxine
GEO: Gene Expression Omnibus
GO: Gene ontology
KEGG: Kyoto Encyclopedia of Genes and Genomes
LAA: Large artery atherosclerosis
MCAO: Middle cerebral artery occlusion
MCODE: Molecular Complex Detection
MF: Molecular function
mGluRs: Metabotropic glutamate receptors
mRS: Modified Rankin Scale
NICD: Notch intracellular domain
NIHSS: National Institutes of Health Stroke Scale NIHSS scoring
NTIS: Non-thyroidal illnesssyndrome
PPI: Protein-protein interaction
PWMH: Periventricular white matter hyperintensity
ROC: Receiver operating characteristic
SD: Standard deviation
SOE: Stroke of other determined etiology
SPSS: Statistical Package for the Social Sciences
STRING: The online Search Tool for the Retrieval of Interacting Genes
SUE: Stroke of undetermined etiology
SVO: Small vessel occlusion
SWMH: Subcortical white matter hyperintensity
T3: Triiodothyronine
T4: Thyroxine
THs: Thyroid hormones
TOAST: Trial of Org 10,172 in Acute Stroke Treatment
TRs: Thyroid receptors
TSH: Thyroid Stimulating Hormone

## References

[1] Zhang D, Gao L, Ye H, Chi R, Wang L, Hu L (2019). Impact of thyroid function on cystatin C in detecting acute kidney injury: a prospective, observational study. BMC Nephrol.

[2] Bunevicius A, Iervasi G, Bunevicius R (2015). Neuroprotective actions of thyroid hormones and low-T3 syndrome as a biomarker in acute cerebrovascular disorders. Expert Rev Neurother.

[3] Iaccarino G, De Rosa M, Trimarco B (2018). Thyroid function predicts increased cardiovascular risk. Int J Cardiol.

[4] de Vries EM, Fliers E, Boelen A (2015). The molecular basis of the non-thyroidal illness syndrome. J Endocrinol.

[5] Lamba N, Liu C, Zaidi H, Broekman M, Simjian T, Shi C (2018). A prognostic role for low tri-iodothyronine syndrome in acute stroke patients: a systematic review and meta-analysis. Clin Neurol Neurosurg.

[6] Wang J, Li F, Xiao L, Peng F, Sun W, Li M (2018). Depressed TSH level as a predictor of poststroke fatigue in patients with acute ischemic stroke. Neurology.

[7] Chen H, Wu Y, Huang G, He W, Lin S, Zhang X (2018). Low tri-iodothyronine syndrome is associated with cognitive impairment in patients with acute ischemic stroke: a prospective cohort study. Am J Geriatr Psychiatry.

[8] Dhital R, Poudel DR, Tachamo N, Gyawali B, Basnet S, Shrestha P (2017). Ischemic stroke and impact of thyroid profile at presentation: a systematic review and meta-analysis of observational studies. J Stroke Cerebrovasc Dis.

[9] Akhoundi FH, Ghorbani A, Soltani A, Meysamie A (2011). Favorable functional outcomes in acute ischemic stroke patients with subclinical hypothyroidism. Neurology.

[10] Baek JH, Chung PW, Kim YB, Moon HS, Suh BC, Jin DK (2010). Favorable influence of subclinical hypothyroidism on the functional outcomes in stroke patients. Endocr J.

[11] Forhead AJ, Fowden AL (2014). Thyroid hormones in fetal growth and prepartum maturation. J Endocrinol.

[12] Sabbaghziarani F, Mortezaee K, Akbari M, Kashani IR, Soleimani M, Hassanzadeh G (2017). Stimulation of neurotrophic factors and inhibition of proinflammatory cytokines by exogenous application of triiodothyronine in the rat model of ischemic stroke. Cell Biochem Funct.

[13] Mokhtari T, Akbari M, Malek F, Kashani IR, Rastegar T, Noorbakhsh F (2017). Improvement of memory and learning by intracerebroventricular microinjection of T3 in rat model of ischemic brain stroke mediated by upregulation of BDNF and GDNF in CA1 hippocampal region. Daru.

[14] Powers WJ, Derdeyn CP, Biller J, Coffey CS, Hoh BL, Jauch EC (2015). 2015 American Heart Association/American Stroke Association focused update of the 2013 guidelines for the early Management of Patients with Acute Ischemic Stroke Regarding Endovascular Treatment: a guideline for healthcare professionals from the American Heart Association/American Stroke Association. Stroke.

[15] Lurbe E, Agabiti-Rosei E, Cruickshank JK, Dominiczak A, Erdine S, Hirth A (2016). 2016 European Society of Hypertension guidelines for the management of high blood pressure in children and adolescents. J Hypertens.

[16] Ross DS, Burch HB, Cooper DS, Greenlee MC, Laurberg P, Maia AL (2016). 2016 American Thyroid Association guidelines for diagnosis and Management of Hyperthyroidism and Other Causes of thyrotoxicosis. Thyroid.

[17] Chamberlain JJ, Rhinehart AS, Shaefer CJ, Neuman A (2016). Diagnosis and Management of Diabetes: synopsis of the 2016 American Diabetes Association standards of medical Care in Diabetes. Ann Intern Med.

[18] Stone NJ, Robinson JG, Lichtenstein AH, Goff DJ, Lloyd-Jones DM, Smith SJ (2014). Treatment of blood cholesterol to reduce atherosclerotic cardiovascular disease risk in adults: synopsis of the 2013 American College of Cardiology/American Heart Association cholesterol guideline. Ann Intern Med.

[19] Arboix A, Morcillo C, Garcia-Eroles L, Oliveres M, Massons J, Targa C (2000). Different vascular risk factor profiles in ischemic stroke subtypes: a study from the "Sagrat Cor Hospital of Barcelona Stroke Registry". Acta Neurol Scand.

[20] Dietary guidelines and the Food Guide Pagoda (2000). The Chinese nutrition society. J Am Diet Assoc.

[21] Adams HJ, Bendixen BH, Kappelle LJ, Biller J, Love BB, Gordon DL (1993). Classification of subtype of acute ischemic stroke. Definitions for use in a multicenter clinical trial. TOAST. Trial of org 10172 in acute stroke treatment. Stroke.

[22] Forini F, Lionetti V, Ardehali H, Pucci A, Cecchetti F, Ghanefar M (2011). Early long-term L-T3 replacement rescues mitochondria and prevents ischemic cardiac remodelling in rats. J Cell Mol Med.

[23] Li J, Donangelo I, Abe K, Scremin O, Ke S, Li F (2017). Thyroid hormone treatment activates protective pathways in both in vivo and in vitro models of neuronal injury. Mol Cell Endocrinol.

[24] Morreale DEG, Obregon MJ, Escobar DRF (2004). Role of thyroid hormone during early brain development. Eur J Endocrinol.

[25] Wang Y, Zhou S, Bao J, Pan S, Zhang X (2017). Low T3 levels as a predictor marker predict the prognosis of patients with acute ischemic stroke. Int J Neurosci.

[26] Vaideeswar P, Singaravel S, Gupte P (2018). The thyroid in ischemic heart disease: an autopsy study. Indian Heart J.

[27] Solter M, Katalinic D, Vargek-Solter V, Dobrovic K, Maric A, Posavec L (2012). Brain tumor as a prototype of severe brain lesion in patients with "low T3 syndrome". Acta Clin Croat.

[28] Scoscia E, Baglioni S, Eslami A, Iervasi G, Monti S, Todisco T (2004). Low triiodothyronine (T3) state: a predictor of outcome in respiratory failure? Results of a clinical pilot study. Eur J Endocrinol.

[29] Lourbopoulos A, Mourouzis I, Karapanayiotides T, Nousiopoulou E, Chatzigeorgiou S, Mavridis T (2014). Changes in thyroid hormone receptors after permanent cerebral ischemia in male rats. J Mol Neurosci.

[30] Warner MH, Beckett GJ (2010). Mechanisms behind the non-thyroidal illness syndrome: an update. J Endocrinol.

[31] Zhang Y, Meyer MA. Clinical analysis on alteration of thyroid hormones in the serum of patients with acute ischemic stroke. Stroke Res Treat. 2010;2010. Article ID 290678. 10.4061/2010/290678 PMC2935184 20847898

[32] Lambertsen KL, Biber K, Finsen B (2012). Inflammatory cytokines in experimental and human stroke. J Cereb Blood Flow Metab.

[33] Malekpour B, Mehrafshan A, Saki F, Malekmohammadi Z, Saki N (2012). Effect of posttraumatic serum thyroid hormone levels on severity and mortality of patients with severe traumatic brain injury. Acta Med Iran.

[34] Ma L, Zhu D, Jiang Y, Liu Y, Ma X, Liu M (2016). Low triiodothyronine: a new facet of inflammation in acute ischemic stroke. Clin Chim Acta.

[35] Wu GH, Kong FZ, Cheng QZ, Luo WF, Du XD (2014). Low T3 syndrome predicts severe neurological deficits of cerebral infarction inpatients with large artery artherosclerosis in internal carotid artery system. Neuro Endocrinol Lett.

[36] Liu J, Wang D, Xiong Y, Yuan R, Tao W, Liu M (2016). Low free triiodothyronine levels are related to symptomatic intracranial hemorrhage and poor functional outcomes after intravenous thrombolysis in acute ischemic stroke patients. Neurol Res.

[37] Lehmann MF, Kallaur AP, Oliveira SR, Alfieri DF, Delongui F, de Sousa PJ (2015). Inflammatory and metabolic markers and short-time outcome in patients with acute ischemic stroke in relation to TOAST subtypes. Metab Brain Dis.

[38] Panaite PA, Barakat-Walter I (2010). Thyroid hormone enhances transected axonal regeneration and muscle reinnervation following rat sciatic nerve injury. J Neurosci Res.

[39] de Souza MS, Romao LF, Faria JC, de Holanda AR, Murray SA, Pellizzon CH (2009). Effect of thyroid hormone T3 on myosin-Va expression in the central nervous system. Brain Res.

[40] Sarkozy G, Griesmaier E, He X, Kapelari K, Urbanek M, Simbruner G (2007). T3 replacement does not prevent excitotoxic cell death but reduces developmental neuronal apoptosis in newborn mice. Eur J Paediatr Neurol.

[41] Sayre NL, Sifuentes M, Holstein D, Cheng SY, Zhu X, Lechleiter JD (2017). Stimulation of astrocyte fatty acid oxidation by thyroid hormone is protective against ischemic stroke-induced damage. J Cereb Blood Flow Metab.

[42] Minato K, Tomimatsu T, Mimura K, Jugder O, Kakigano A, Kanayama T (2013). Hypoxic preconditioning increases triiodothyronine (T3) level in the developing rat brain. Brain Res.

[43] Da CR, Laureano-Melo R, Oliveira KC, de Carvalho MM, Kasamatsu TS, de Barros MR (2016). Antidepressant behavior in thyroidectomized Wistar rats is induced by hippocampal hypothyroidism. Physiol Behav.

[44] Popa-Wagner A, Buga AM, Kokaia Z (2011). Perturbed cellular response to brain injury during aging. Ageing Res Rev.

[45] Dezonne RS, Stipursky J, Araujo AP, Nones J, Pavao MS, Porcionatto M (2013). Thyroid hormone treated astrocytes induce maturation of cerebral cortical neurons through modulation of proteoglycan levels. Front Cell Neurosci.

[46] Skowrońska Katarzyna, Obara-Michlewska Marta, Zielińska Magdalena, Albrecht Jan (2019). NMDA Receptors in Astrocytes: In Search for Roles in Neurotransmission and Astrocytic Homeostasis. International Journal of Molecular Sciences.

[47] Takenouchi T, Hashida N, Torii C, Kosaki R, Takahashi T, Kosaki K (2014). 1p34.3 deletion involving GRIK3: further clinical implication of GRIK family glutamate receptors in the pathogenesis of developmental delay. Am J Med Genet A.

[48] Stojanovic T, Capo I, Aronica E, Adle-Biassette H, Hoger H, Sieghart W (2016). The alpha1, alpha2, alpha3, and gamma2 subunits of GABAA receptors show characteristic spatial and temporal expression patterns in rhombencephalic structures during normal human brain development. J Comp Neurol.

[49] Riccio O, Hurni N, Murthy S, Vutskits L, Hein L, Dayer A (2012). Alpha2-adrenergic receptor activation regulates cortical interneuron migration. Eur J Neurosci.

[50] Olson KE, Kosloski-Bilek LM, Anderson KM, Diggs BJ, Clark BE, Gledhill JJ (2015). Selective VIP receptor agonists facilitate immune transformation for dopaminergic Neuroprotection in MPTP-intoxicated mice. J Neurosci.

[51] Cui D, Shang H, Zhang X, Jiang W, Jia X (2016). Cardiac arrest triggers hippocampal neuronal death through autophagic and apoptotic pathways. Sci Rep.

